# Contextual effects in musculoskeletal pain: are we overlooking essential factors?

**DOI:** 10.3389/fpsyg.2025.1537242

**Published:** 2025-02-17

**Authors:** David Poulter, Alvisa Palese, Lia Rodeghiero, Elisa Carlino, Jorge E. Esteves, Chad Cook, Giacomo Rossettini

**Affiliations:** ^1^MT3 Clinical Education and Consulting, Coon Rapids, MN, United States; ^2^Department of Medical Sciences, University of Udine, Udine, Italy; ^3^Department of Rehabilitation, Hospital of Merano (SABES-ASDAA), Teaching Hospital of Paracelsus Medical University (PMU), Merano-Meran, Italy; ^4^Department of Neuroscience “Rita Levi Montalcini,” University of Turin, Turin, Italy; ^5^Malta ICOM Educational, Gzira, Malta; ^6^Clinical-Based Human Research Department, Foundation COME Collaboration, Pescara, Italy; ^7^Escola Superior de Saúde Atlântica, Barcarena, Portugal; ^8^Department of Orthopedics, Duke University, Durham, NC, United States; ^9^Duke Clinical Research Institute, Duke University, Durham, NC, United States; ^10^Department of Population Health Sciences, Duke University, Durham, NC, United States; ^11^School of Physiotherapy, University of Verona, Verona, Italy; ^12^Musculoskeletal Pain and Motor Control Research Group, Faculty of Sport Sciences, Universidad Europea de Madrid, Madrid, Spain

**Keywords:** expectation, placebo effects, nocebo effects, physiotherapy, nursing, chiropractic, osteopathy, pain

## 1 Introduction

The role of contextual effects in musculoskeletal pain research and clinical practice has garnered growing interest in recent years (Rossettini et al., 2024). While ongoing research has advanced knowledge, it has also sparked debate between clinicians, clinical trialists and other researchers (Saueressig et al., 2024a; Ezzatvar et al., 2024a; Saueressig et al., 2024b; Ezzatvar et al., 2024b). The controversy over contextual effects in musculoskeletal pain research and practice highlights the tension between leveraging their therapeutic potential and minimizing them to preserve treatment specificity (Keter et al., 2025).

Clinicians value any factor that improves patient outcomes, while clinical trialists seek to minimize contextual effects to preserve treatment specificity (Sherriff et al., 2023). Mechanistic researchers, focused on how these factors affect our brain and behavior, struggle to quantify them while excluding them from research protocols and clinical trials (Kamper and Williams, 2013). This tension intensifies in non-pharmacological interventions (Hohenschurz-Schmidt et al., 2023a,b). Additionally, the varying influence of contextual factors on treatment outcomes (Saueressig et al., 2024c), underscores the need for further research to optimize their role across clinical settings and interventions.

Differing perspectives, while enriching scientific debate, may create divisions among professionals, distancing them from the shared goal of leveraging contextual effects to benefit patients with musculoskeletal disorders. Contextual effects are defined as those generated by CFs in clinical practice (Di Blasi et al., 2021). An international consensus recently described CFs as “components of all therapeutic encounters,” including patient, clinician, treatment, patient-clinician relationship, and encounter context characteristics (Cook et al., 2023).

This opinion paper aims to foster dialogue and bridge the gap between clinical practice and research, creating a foundation for collaboration and mutual understanding. First, we examine potential sources of misunderstanding about contextual effects leading to conflicts. Then, we discuss their implications for clinical and research fields. Different professionals (clinicians and researchers) from different disciplines (nursing, psychology, physical therapy, osteopathy, epidemiology) have been involved to ensure a multidisciplinary, inclusive approach, valuing all perspectives.

## 2 Discussion

**Figure 1:**
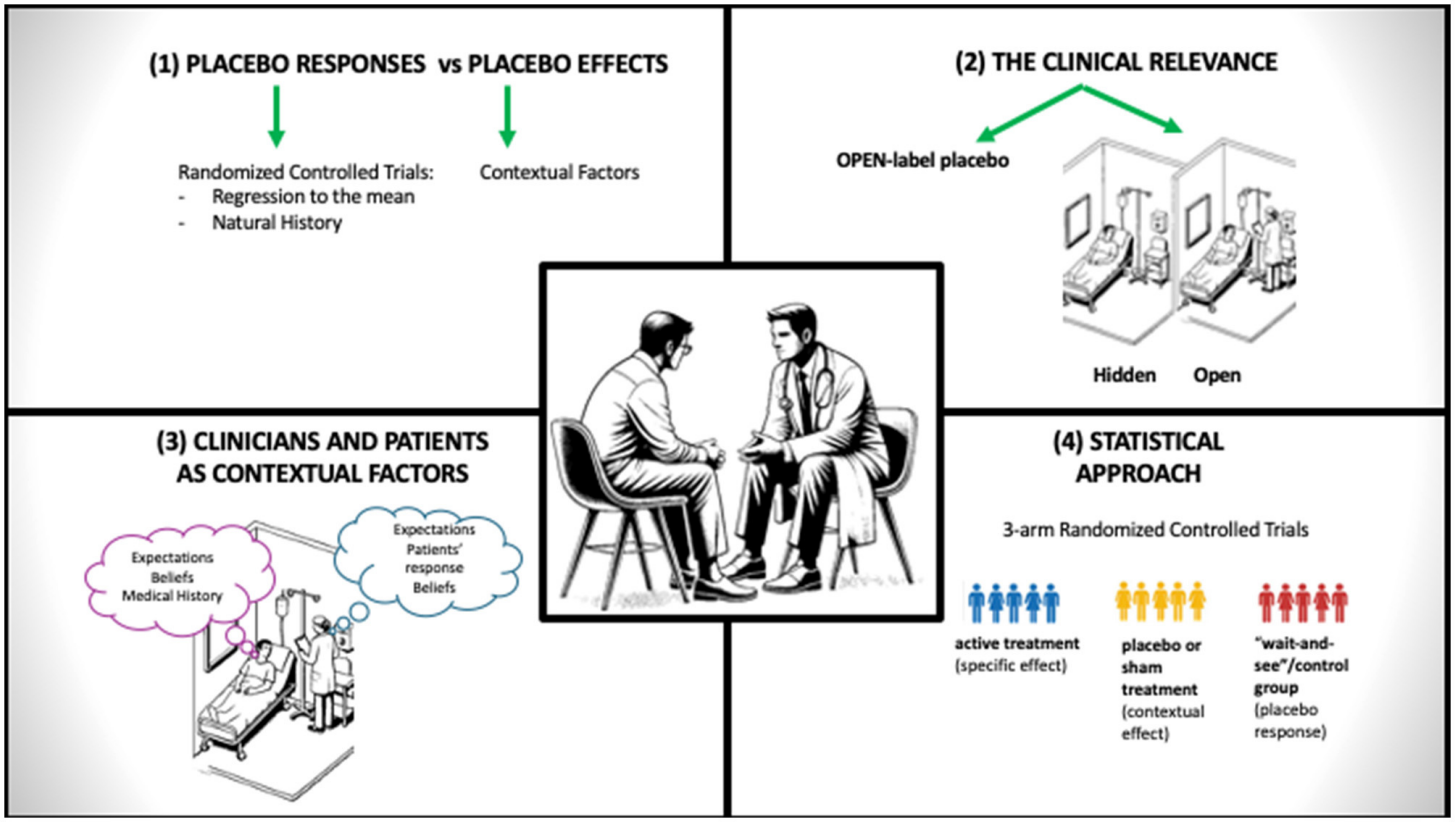
Sources of misunderstanding discussing contextual effects. This diagram highlights the different possible sources of misunderstanding between clinicians and researchers when discussing contextual effects in musculoskeletal care.

Potential conflicts regarding contextual effects between clinicians and researchers may stem from misunderstandings in four areas: (1) terms used to define them, (2) their clinical relevance during placebo use, (3) their value in musculoskeletal pain treatments, and (4) statistical methods to estimate their effect (Figure 1).

### 2.1 The first source of misunderstanding—terminology

The diverse terminology in scientific literature, such as “placebo effects” and “placebo responses,” often used interchangeably or confused with contextual effects, is a significant source of misunderstanding. Contextual effects are linked with placebo effects in mechanistic studies exploring biological and psychosocial pathways, while placebo responses are not (Evers et al., 2018; Cashin et al., 2021).

A “placebo” is an inert substance or intervention used in randomized controlled trials (RCTs) as a control for testing treatments. “Placebo responses” refer to health improvements in the placebo arm of an RCT, compared with the active treatment arm. A placebo study mimics the intervention being tested without producing its mechanistic benefits and is ideally double-blind, including socio-emotional cues (contextual cues) during patient-practitioner interactions to elicit contextual effects.

Standard double-blind placebo-controlled trials lack a natural history control group. Thus, improvements in the placebo arm may reflect factors like regression to the mean or the natural course of the condition, rather than neurobiological changes from expectations or learning, hallmarks of placebo effects (Carlino and Vase, 2018). Regression to the mean occurs when extreme values normalize over time due to random variability (Sean et al., 2024). Natural resolution refers to spontaneous improvement in conditions that self-resolve, like wound healing, fracture healing, or fluctuating chronic pain (Sean et al., 2024).

“Placebo effects” arise from psychosocial factors surrounding therapy (Saueressig et al., 2024c), involving neurotransmitter release (e.g., opioids, cannabinoids, dopamine) and changes in brain regions like the prefrontal cortex, amygdala, and periaqueductal gray (Wager and Atlas, 2015). Placebo effects involve positive use of contextual factors, while nocebo effects result from their negative application (Carlino et al., 2016). Both effects influence outcomes of musculoskeletal pain treatments (e.g., injections, surgery, exercise, massage) by integrating with specific treatment components to shape clinical results and patient experiences (Rossettini et al., 2023).

While some researchers and clinicians recognize these distinctions, the broader challenge is the complexity and variability in how these terms are understood, applied, and interpreted across different contexts. This inconsistency affects research design, data interpretation, and communication, fuelling conflicts among researchers, clinicians, and trialists with differing priorities.

### 2.2 The second source of misunderstanding—the clinical relevance

A second source of misunderstanding involves the consideration of how placebo treatments (e.g., placebo/sham pills and surgeries), although inherently inert and lacking therapeutic effect, can still influence patient symptoms through priming induced by contextual effects. This has been demonstrated in clinical studies using open-label placebo pills and open-hidden paradigm.

An example of contextual priming is open-label placebo injections use. In one study, chronic back pain patients received saline injections explicitly labeled as placebos, alongside information on their potential pain relief, in addition to usual care (Ashar et al., 2024). Clinicians explained the placebo effect, using Pavlov's dogs as an analogy, suggesting the body could respond automatically to a neutral stimulus—the placebo injection. This is a clear example of contextual priming, where any observed clinical effect arises not from the injection itself but from the clinician's explanation, which drives the modest therapeutic response.

The clinical relevance of CFs emerges from studies using the open-hidden approach, administering treatment in two ways (Benedetti et al., 2011). In the open condition, patients know they are receiving treatment, often accompanied by verbal cues from the provider, activating expectations and beliefs to enhance the placebo effect. In the hidden condition, treatment is delivered without the patient's awareness, often via automated means. This clarifies how much of a treatment's efficacy is due to its active vs. psychological components. Despite its limitation to immediate or short-term effects, this approach also shows that contextual effects occur even with active treatments, highlighting that a treatment's effectiveness combines both active and contextual components (Benedetti et al., 2011).

### 2.3 The third source of misunderstanding—beliefs and expectations

A third source of misunderstanding arises from the belief that CFs are solely linked to therapeutic intervention itself. This view is partly inaccurate, as clinicians—through their beliefs, knowledge, and behavior—and patients—through their expectations and clinical history—are also active components of the context, embodying contextual elements themselves.

The clinicians' beliefs and empathy can generate contextual effects during the caring for patients with musculoskeletal pain. This lack of equipoise effect is, for example, evident in a study on spinal manipulation and pain relief, where clinicians who strongly believed in the treatment's efficacy and showed a clear preference for it increased the likelihood of patients experiencing beneficial pain relief by 68.3 times compared to treatments provided by neutral clinicians (Bishop et al., 2017). Evidence also highlights the crucial role of clinician empathy in achieving positive outcomes. For example, a study on adults with chronic low back pain demonstrated that patients treated by highly empathetic clinicians experienced significantly better outcomes in pain relief, functionality, and overall quality of life over 12 months (Licciardone et al., 2024). These results emphasize how clinician attitudes and empathy can activate contextual effects, significantly influencing clinical outcomes. Empathetic encounters foster trust and enhance the patient-clinician relationship, reinforcing that effective communication and empathy are key to strong therapeutic alliances and better patient outcomes (Wang et al., 2022).

Patient expectations can trigger contextual effects. In a neck pain study, patients who expected cervical manipulation to help (positive expectations) but did not receive it, had significantly lower odds of treatment success (OR = 0.16; 95% CI: 0.04, 0.72) than those who both expected and received it. Among those treated, patients with positive expectations reported significantly less disability than those without (mean difference: −3.8; 95% CI: −5.9, −1.5; *P* = 0.006; Bishop et al., 2013). This suggests that expectations can independently impact outcomes, functioning separately from the direct effects of the treatment itself.

The dynamic interplay between patient expectations and their individualized responses to specific treatments, combined with clinician beliefs and decision-making processes, introduces an additional layer of complexity. In a study examining treatment decisions in clinical practice, physicians were significantly more likely to administer placebo treatments to patients who appeared responsive to them (Piedimonte et al., 2024). This finding highlights that physicians' treatment choices are influenced by patient responsiveness, while patient responsiveness is, in turn, shaped by clinician characteristics and decision-making processes.

### 2.4 The fourth source of misunderstanding—the statistic conundrum

A fourth potential source of misunderstandings stems from the use of varying statistical methods to estimate contextual effects in meta-analyses, along with the differing epistemological assumptions that guide these analyses.To accurately estimate contextual effects, meta-analyses should include three-arm randomized controlled trials (RCTs). In such trials, one group receives the active treatment (specific effect), another receives a placebo or sham treatment (contextual effect), and a third “wait-and-see” control group accounts for natural progression and regression to the mean (placebo response; Cashin et al., 2021). However, despite support in the literature (Saueressig et al., 2024d; Hohenschurz-Schmidt et al., 2024), such meta-analyses are often impractical, particularly in fields using non-pharmacological treatments (e.g., rehabilitation), where including a no-treatment control group can raise ethical concerns (Cashin et al., 2021). A possible approach, known as Delayed Treatment Design, involves administering the active non-pharmacological treatment at different time points, which avoids ethical concerns and enables comparisons between the treatment's active effects and natural symptom fluctuations (Mongini et al., 2012). Alternative approaches, like the Proportion Attributable to Contextual Effects (Zou et al., 2016), have also been proposed. While commonly used to estimate contextual effects in musculoskeletal pain treatments (de Roode et al., 2024; Ezzatvar et al., 2024c), these methods have limitations that can overestimate effects and introduce bias (for more details see: Saueressig et al., 2024c).

Researchers should recognize the challenges of measuring CFs, as the validity of a simple additive response model has been called into question. This complexity arises from the diverse responses to contextual manipulations and individual differences among participants. Three distinct response types have been identified: antagonistic, synergistic, and reversal (qualitative interactions). In an antagonistic interaction, the treatment effect is less than the combined placebo and specific effects. Conversely, in a synergistic model, the treatment effect surpasses the sum of the placebo and specific effects. This suggests that contextual manipulation can either diminish or enhance the specific effect of treatment. In reversal cases, a nocebo effect may negate the specific effect—for example, when pain is felt despite the application of a topical analgesic under nocebo-informed conditions (Boussageon et al., 2022).

### 2.5 All is not lost: implications for clinicians and researchers

Despite possible misunderstandings, we believe there remains significant ground to explore, and it is premature to conclude discussions on contextual effects in musculoskeletal pain. We propose that the clinical and research communities can find common ground and overcome tension through constructive dialogue and active involvement in organizations dedicated to studying these effects (Rossettini et al., 2024). In this context, the Society for Interdisciplinary Placebo Studies (SIPS) has provided numerous opportunities for sharing knowledge, building connections and planning studies through roundtables and panels, as demonstrated by the four international conferences held thus far (SIPS Conferences, 2024).

Clinicians should communicate the challenges they face in managing patients with musculoskeletal pain, particularly when utilizing contextual effects to enhance placebo benefits and mitigate nocebo effects (Table 1). Additionally, clinicians should recognize that everyday clinical practice differs significantly from RCTs, as it is uncontrolled and influenced by unforeseen factors that can affect clinical outcomes. Consequently, careful interpretation of evidence and thoughtful translation of findings into real-world practice are essential.

**Table 1:** Contextual effects in musculoskeletal pain: questions unresolved.

**Reflections for future studies**
• Which CFs most influence patient outcomes?• How do different CFs interact to influence patient outcomes?• Are CFs more significant in certain phases of pain management (e.g., history taking, physical examination, therapeutic administration)?• Do CFs act differently in acute vs. chronic conditions?• Do CFs impact differently in presence of nociceptive, nociplastic, and neuropathic pain?• Does the effect of CFs on therapeutic outcomes vary in the short, medium, and long term?• Do CFs affect subjective and objective/physiological outcomes in the same way?• Is the effect of CFs affected by social, cultural, religious, economic and geopolitical influences?• How do technological advancements (e.g., telehealth, digital devices, artificial intelligence) interact with CFs to influence outcomes?• Are CFs more impactful in self-managed vs. clinician-guided treatments?• Do CFs vary in their impact across different pain management modalities (e.g., pharmacological vs. non-pharmacological)?• Are there CFs that consistently predict positive vs. negative outcomes across different populations?• Are there universal CFs applicable across different healthcare systems and geopolitical settings?• Are CFs stable over time, or do they evolve during the course of a condition?• Do CFs differ in their impact between early-stage vs. advanced-stage conditions?• How do CFs influence the cost-effectiveness of treatment interventions?• Do CFs have different influences on passive treatments (e.g., injections and physical modalities) and active treatments (e.g., therapeutic exercises and physical activities)?

CF, contextual factors.

Researchers, on the other hand, can support clinicians by producing scientifically robust research on contextual effects that mirrors the conditions of everyday clinical practice while maintaining the necessary rigor. In this context, real-world RCTs, planned to follow the Consolidated Standards of Reporting Trials (CONSORT) guidelines and conducted outside laboratories, where multiple CFs are applied simultaneously during the administration of evidence-based treatment, may ensure both internal and external validity (Andreu et al., 2024). Additionally, researchers designing placebo RCTs should detail the CFs present in both the active intervention and placebo groups, following recommendations for the development, implementation, and reporting of control interventions in efficacy and mechanistic trials of physical, psychological, and self-management therapies (CoPPS Statement) to ensure a thorough and balanced description of these effects (Hohenschurz-Schmidt et al., 2023c).

## 3 Conclusion

In summary, CFs are integral and unavoidable components of everyday musculoskeletal pain management, making them inseparable from clinical practice. As the scholarly debate evolves, our advice to clinicians regarding these effects echoes the timeless lyrics: “Accentuate the positive, eliminate the negative, and latch on to the affirmative” (Mercer, 1944), adopting empathy, validation, and a patient-centered communication style during clinical encounters. Meanwhile, researchers are tasked with the challenge of disentangling the “contextual effects cake.” While this perspective does not serve as the final word on contextual effects, we hope it offers a step toward bridging the gap between clinical practice and research. Ultimately, fostering collaboration between these domains has the potential to deliver meaningful benefits for patients with musculoskeletal pain.
